# Artificial intelligence application versus physical therapist for squat evaluation: a randomized controlled trial

**DOI:** 10.1038/s41598-021-97343-y

**Published:** 2021-09-13

**Authors:** Alessandro Luna, Lorenzo Casertano, Jean Timmerberg, Margaret O’Neil, Jason Machowsky, Cheng-Shiun Leu, Jianghui Lin, Zhiqian Fang, William Douglas, Sunil Agrawal

**Affiliations:** 1 Vagelos College of Physicians and Surgeons, Columbia University, New York, NY USA; 2 NewYork-Presbyterian/Columbia University Irving Medical Center, New York, NY USA; 3 Columbia University Irving Medical Center, New York, NY USA; 4 Hospital for Special Surgery, New York, NY USA; 5 Department of Biostatistics, Columbia University, New York, NY USA; 6 Department of Psychiatry, University of Hong Kong, Pok Fu Lam, Hong Kong; 7 Departments of Mechanical Engineering and Rehabilitation Medicine, Columbia University, 500 W. 120th Street #510, New York, NY 10027 USA

**Keywords:** Translational research, Rehabilitation

## Abstract

Artificial intelligence technology is becoming more prevalent in health care as a tool to improve practice patterns and patient outcomes. This study assessed ability of a commercialized artificial intelligence (AI) mobile application to identify and improve bodyweight squat form in adult participants when compared to a physical therapist (PT). Participants randomized to AI group (n = 15) performed 3 squat sets: 10 unassisted control squats, 10 squats with performance feedback from AI, and 10 additional unassisted test squats. Participants randomized to PT group (n = 15) also performed 3 identical sets, but instead received performance feedback from PT. AI group intervention did not differ from PT group (log ratio of two odds ratios =  − 0.462, 95% confidence interval (CI) (− 1.394, 0.471), p = 0.332). AI ability to identify a correct squat generated sensitivity 0.840 (95% CI (0.753, 0.901)), specificity 0.276 (95% CI (0.191, 0.382)), PPV 0.549 (95% CI (0.423, 0.669)), NPV 0.623 (95% CI (0.436, 0.780)), and accuracy 0.565 95% CI (0.477, 0.649)). There was no statistically significant association between group allocation and improved squat performance. Current AI had satisfactory ability to identify correct squat form and limited ability to identify incorrect squat form, which reduced diagnostic capabilities.

**Trial Registration** NCT04624594, 12/11/2020, retrospectively registered.

## Introduction

Artificial intelligence technology (AI) is a general term to describe computers exhibiting human-like intelligence and reason^1^. AI is becoming more prevalent in health care as a tool to improve practice patterns and patient outcomes. AI holds promise in that it can be used to create programs to replicate complex cognitive tasks to assist clinicians in patient management. Examples include analysis of imaging data for diagnosis of cancer and heart disease; extraction of unstructured data from electronic medical records for evaluation; and creation of socially assistive robot exercise coaching for older adults^1–3^. These examples demonstrate that AI can be integrated into software systems such as electronic medical records and hardware systems such as robots or devices.

In this study, AI is used to examine bodyweight exercise performance. Benefits of exercise impact multiple areas of health and disease including dementia risk, cardiovascular and musculoskeletal disorders, and obesity^4–7^. The AI exercise mobile application (app) used for this research is built with patent pending motion tracking technology which monitors and provides real-time audiovisual feedback on a person’s exercise performance. The technology relies on mobile phone video capture capability and does not require any additional equipment.

This app benefits from independent operation. Users do not need another person to control the app, and the user does not need to wear additional sensors. Other applications may provide audiovisual instructions, but do not provide corrective feedback when exercises are performed incorrectly^8^. Additionally, a previous trial demonstrated the effectiveness of the AI app in treating lower back pain^9^. This trial tested how the app identifies and improves bodyweight squats when compared to feedback from a physical therapist since this particular exercise is considered a foundational and compound movement used in activities of daily living^10,11^.

## Methods

### Study design

In this randomized, blinded, controlled trial, 30 participants were randomly assigned to either the AI (n = 15) or PT (n = 15) group. The local institutional review board approved the study, and all participants provided written informed consent. Trial number NCT04624594 retrospectively registered on 12/11/2020 (See Supplementary Files 1 and 2).

### Participants

Research population was academic institution affiliates, age 20–35, without any preexisting medical condition that precluded them from participating in bodyweight exercise for 10 min. Participants could withdraw at any time and were not paid to be in the study. Person-to-person recruitment and flyers were used in October 2019. Participants were randomly assigned to either the AI or PT group in a 1:1 ratio using the random choice selection function in Excel. Participants were also assigned a unique identifier number to sign up for a time slot. Both the AI and PT groups had 7 female and 8 male participants.

### Squat definition

Based on pre-existing squat literature descriptions and published squat best practices^12–18^, the PT and three independent evaluators collectively agreed on this study’s official squat definition:

Individual starts in a standing position with feet flat on the floor, knees and hips in a neutral, extended anatomical position, spine in an upright position with preservation of its natural curves, and hands held in front of body. Squat movement begins with descent phase initiated by “sitting back” as hips, knees, and ankles flex simultaneously. Individual should descend until hip joint becomes level with knee joint, without letting the knees extend past toes. Ascent is achieved through simultaneous extension of the hips, knees, and ankles, continuing until the subject has returned to starting position.

### Intervention

On assigned day and time, each participant reported to campus research space individually for 15 min to maintain anonymity. The first 5 min were reserved for a presentation of key information about the trial. Participants had the opportunity to discuss the information before volunteering their written informed consent.

In the subsequent 10 min, participants were observed by the AI (operating from an iPhone X) and PT from the sagittal right plane 3 m away. A video camera simultaneously recorded the exercises at this same position. Additional supervising researcher was always present for added safety. For standardization, all participants were instructed to keep hands in front of their body, squat until knees flexed to 90 degrees, and maintain a cadence of 2 s for the descent phase and two seconds for the ascent phase.

The PT providing feedback had more than a decade of training and practice in neurorehabilitation and extensive experience training athletes. The PT was provided a standardized list of corrections based on the common AI evaluations, and was also free to provide any necessary feedback not contained in the list. The common AI evaluations included: body leaning too far to the front, not squatting deep enough (< 90°), squatting too deep (> 90°), knees extending past toes, neck extended too far upwards, neck flexed too far downwards, motion was too fast, and motion was too slow.

All participants performed 10 bodyweight squat “control” repetitions without feedback followed by one minute of rest. Those in the AI group then performed 10 more “practice” repetitions with real-time audiovisual feedback from the app followed by 1 min of rest. The AI’s design provided one piece of feedback, if necessary, with a voice statement and on-screen video per repetition (e.g. when participant performed squat repetition with their neck flexed downward, AI suggested keeping their head up with on-screen instruction). Those in the PT group (n = 15) also performed 10 “practice” repetitions with one piece of feedback per repetition, if necessary, from the PT. Participants in both groups then performed 10 “test” repetitions without feedback.

### Outcomes

Filmed repetitions from the “control” and “test” sets were scored by three independent evaluators as correct or incorrect. They were blinded to participant group and squat set. If repetition was incorrect, a one sentence justification was provided; they used the aforementioned standardized correction list as a guide and were free to make other form corrections they deemed necessary. If at least two panel evaluators scored a squat as correct, the “majority score” was correct, and vice versa for incorrect squats. The outcome of the intervention for a given participant was considered a “success” if participant had more correct than incorrect squats after intervention.

### Statistical analysis

Graphical presentation and descriptive statistics were generated to show the frequencies and percentages of correct and incorrect squats in control and test sets as scored by AI and each evaluator. In addition, AI and evaluators also provided one piece of feedback per incorrect squat. Evaluators identified 8 additional corrections in addition to the standardized list: arms not out in front of chest, asymmetrical weightbearing, incomplete extension on ascent, early lumbar spine motion, body leans too far back, trunk folds or torso bends, knee dominant movement, and torso-initiated movement. Graphical presentation and descriptive statistics were presented to report the feedback provided by AI and evaluators for incorrect squats.

Majority scores were used as the gold standard to calculate sensitivity (AI ability to identify a correct squat), specificity (AI ability to identify an incorrect squat), positive predictive value (PPV), negative predictive value (NPV), and accuracy of the scores determined by AI. Furthermore, each evaluator was also considered as the gold standard to examine the performance of AI. Generalized Estimating Equations (GEE) were used to account for within subject correlation due to repeated measures when calculating the 95% confidence intervals for the above operating characteristics.

A generalized linear model was used to compare the change over time of the probability of doing correct squats between the AI and PT interventions. GEE with exchangeable correlation structure was employed to account for within subject correlations^19^. The statistical model included the intercept, post-intervention indicator (vs. pre-intervention), AI group indicator (vs. PT group), and the interaction between the two indicators. Gender was also included in the model to adjust for potential confounding. The regression coefficient corresponding to the interaction term represented the log ratio of two odds ratios and allowed comparison of AI and PT group intervention effect. The odds of “success” (i.e., more correct than incorrect squats after intervention) were compared between AI and PT group via logistic regression analysis. In addition, Light’s Kappa was used to evaluate inter-rater reliability of the three evaluators^20^. Findings were declared statistically significant if p ≤ 0.05. Analyses were performed in RStudio^21^.

### Sample size calculation

The study was designed to detect a 40-percentage point difference (i.e., 65% vs. 25%, which we considered as a clinically important difference) with 80% power for a two-sided 0.05-level test. We conducted the sample size calculation assuming that each participant would complete 20 squats, having a within subject correlation (i.e., intra-subject correlation ICC) no greater than 0.7. The above calculation led to a recruitment of 15 subjects per group (30 in total) and required data with 300 observations per group (600 in total).

### Ethics approval

Columbia University institutional review board approved study protocol AAAS7301 on October 15, 2019, which was performed in accordance with the standards defined in the 1964 Declaration of Helsinki.

### Informed consent

Informed consent was obtained from all individual participants included in this study.

## Results

### Descriptive statistics

**Figure 1:**
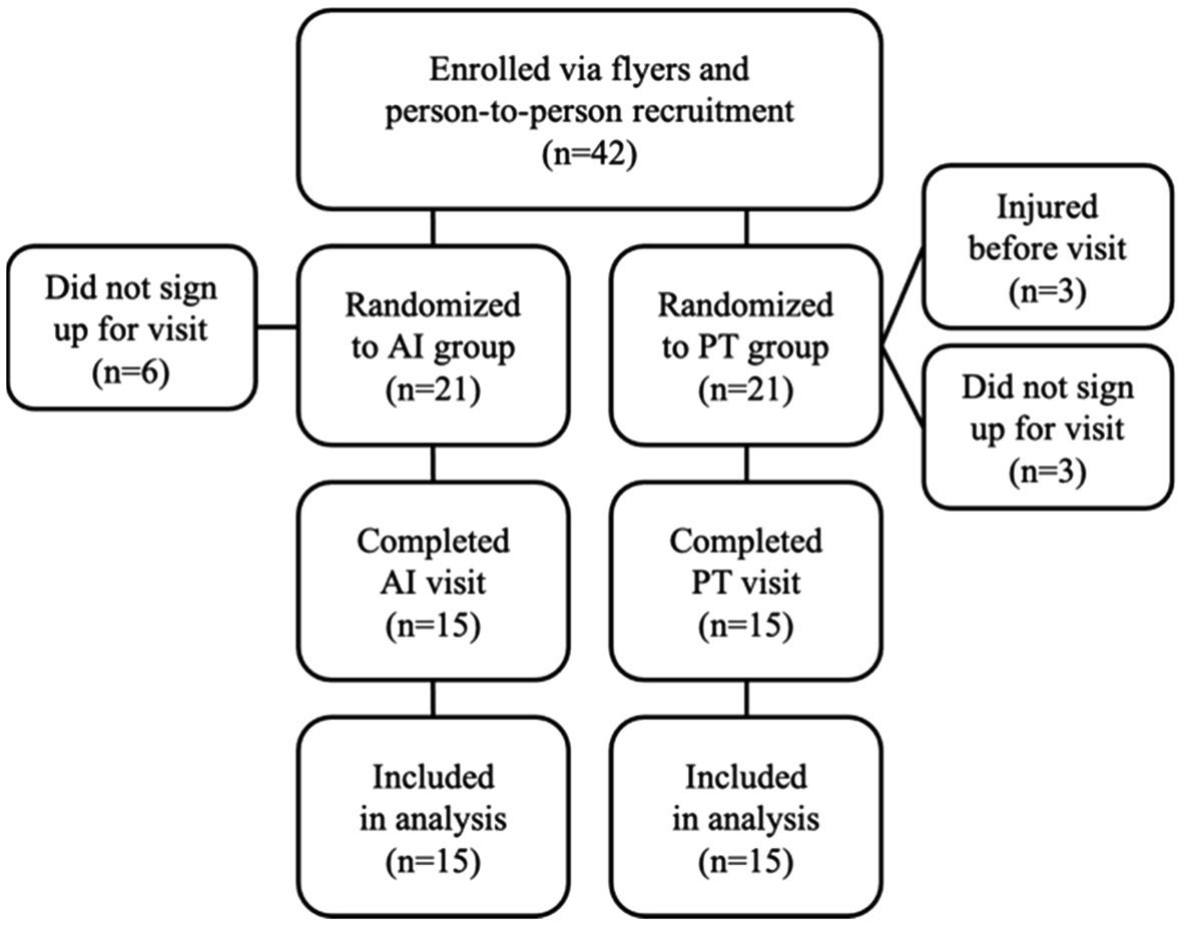
Participant flowchart. 42 people were eligible, but 6 people did not sign up for a time slot and 3 people were injured prior to participation.

**Figure 2:**
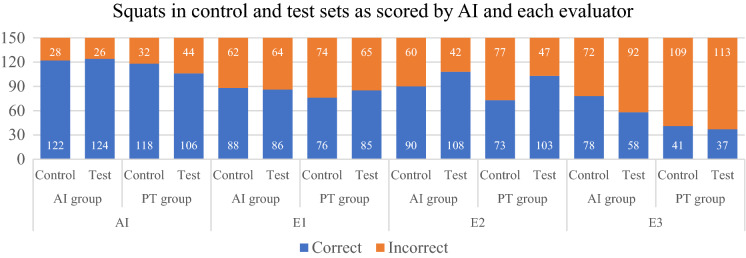
Correct and incorrect squats as scored by AI and evaluators (E1 = Evaluator 1, E2 = Evaluator 2, E3 = Evaluator 3). “Control” refers to the first set of 10 unassisted squat repetitions. “Test” refers to the third and last set of 10 unassisted squat repetitions performed by participants after receiving feedback in the second set.

**Figure 3:**
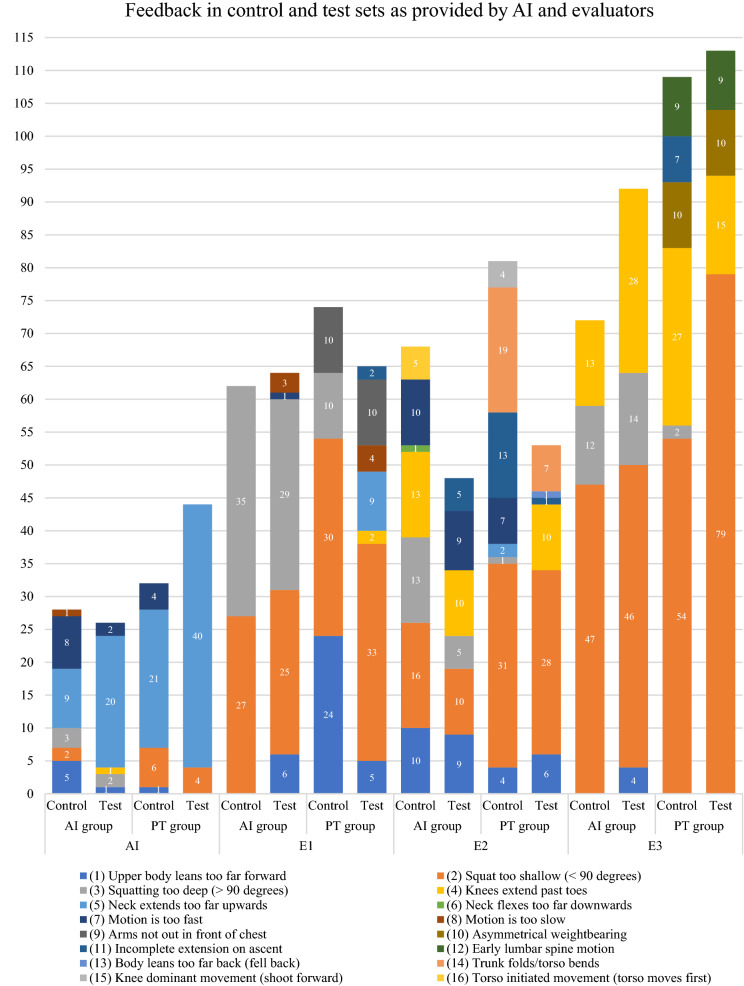
Feedback for incorrect squats as provided by AI and evaluators (E1, E2, E3).

Data for 30 participants were collected (see Fig. 1). Correct and incorrect squats were tabulated for AI and the three evaluators (see Fig. 2). Of the 600 squat repetitions performed in the control and test sets, 307 (51.2%) received a majority score of correct and 293 (48.8%) a majority score of incorrect. The three evaluators completely agreed for 294 (49%) repetitions: 155 (25.8%) squats scored as correct and 139 (23.2%) squats scored as incorrect. The most common feedback provided by 2/3 evaluator majority was “squat too shallow (< 90 degrees)” (11.3%). The most common feedback provided by AI was “neck extends too far upwards” (15%) (see Fig. 3).

### Sensitivity, specificity, PPV, NPV, and accuracy

Operating characteristics of AI were reported with their 95% confidence intervals (see Table 1). AI was also compared with instances where panel of evaluators had 3/3 complete agreement instead of 2/3 majority agreement: sensitivity was 0.865 (95% CI (0.751, 0.931)), specificity = 0.281 (95% CI (0.179, 0.411)), PPV = 0.573 (95% CI (0.390, 0.737)), NPV = 0.650 (95% CI (0.370, 0.855)), and accuracy = 0.588 (95% CI (0.466, 0.701)).

**Table 1:** Operating characteristics and 95% confidence intervals for AI versus evaluators.

	AI vs. majority (control + test set)		AI vs. majority (control set)		AI vs. majority (test set)		AI vs. E1 (control + test set)		AI vs. E2 (control + test set)		AI vs. E3 (control + test set)	
	Estimate	95% CI	Estimate	95% CI	Estimate	95% CI	Estimate	95%CI	Estimate	95% CI	Estimate	95% CI
Sensitivity	0.840	0.753–0.901	0.871	0.770–0.931	0.812	0.697–0.891	0.812	0.727–0.875	0.826	0.746–0.885	0.846	0.748–0.910
Specificity	0.276	0.191–0.382	0.268	0.178–0.382	0.286	0.181–0.421	0.253	0.179–0.344	0.288	0.192–0.407	0.251	0.177–0.343
PPV	0.549	0.423–0.669	0.533	0.389–0.672	0.565	0.426–0.695	0.579	0.464–0.685	0.657	0.531–0.765	0.385	0.264–0.522
NPV	0.623	0.436–0.780	0.683	0.482–0.834	0.571	0.356–0.763	0.515	0.372–0.656	0.500	0.323–0.677	0.746	0.550–0.876
Accuracy	0.565	0.477–0.649	0.563	0.458–0.663	0.567	0.467–0.662	0.565	0.488–0.639	0.623	0.537–0.703	0.463	0.380–0.549

“AI vs. Majority” compares AI with panel majority of the 3 evaluators: E1, E2, and E3. “Control” refers to the first set of 10 unassisted squat repetitions. “Test” refers to the third and last set of 10 unassisted squat repetitions performed by participants after receiving feedback in the second set.

### Comparison of AI versus PT intervention effects

Findings from the GEE analysis suggested that AI group intervention effect, in terms of change over time in the probability of a correct squat pre- versus post-intervention, did not differ from the PT group (log ratio of two odds ratios =  − 0.462, 95% CI (− 1.394, 0.471), p = 0.332). Proportion of participants with more correct squats after the intervention was greater in the PT group, but was not statistically significant at the 0.05 level (PT vs. AI: 60% vs. 27%, odds ratio = 4.125, 95% CI (0.883, 19.273), p = 0.072).

### Inter-rater reliability

Light’s Kappa (weighted average of Cohen’s Kappa for each evaluator pair) for inter-rater reliability of the three evaluators scoring 600 squat repetitions was 0.337. Cohen’s Kappa for evaluator 1 and 2, 1 and 3, and 2 and 3 were 0.320, 0.266, and 0.319, respectively. In the subset of squats determined to be incorrect by 2/3 panel majority, Light’s Kappa for inter-rater agreement on the feedback provided for these incorrect squats was 0.407 (Supplementary Material).

## Discussion

This trial was an independent university medical center evaluation of commercialized private sector technology to study the ability of an AI exercise mobile application to identify and improve bodyweight squat form in 30 adult participants. The GEE analysis revealed no statistically significant difference between AI and PT group on squat performance. While not statistically significant at p < 0.05, trends of these analyses suggested that PT intervention may have had favorable effects on squat improvement; PT group had above 4 times greater odds of having more correct squats when compare to AI group. Lack of statistical power may have been an issue for such an effect not attaining the 0.05 level of statistical significance.

The AI had satisfactory ability to identify correct squat form as evidenced by its sensitivity values (see Table 1) which are comparable with each individual evaluator and the collective panel^22^. Conventional motion-tracking systems use multiple high-speed cameras with ground force plates or wearable inertial measurement units^23^. When comparing the present AI data to previous studies validating these conventional systems, the AI sensitivity matched or exceeded these systems for squat movements^23–28^. When compared with existing systems, a clear benefit of the AI mobile application is the absence of complex machinery or expensive wearable sensors for functioning. However, the AI specificity to identify incorrect squats was insufficient and attributed to the low accuracy of 0.565 (95% CI 0.524–0.605).

Descriptive statistics indicated possible factors contributing to the low accuracy. AI only identified squats that were deemed too shallow (< 90 degrees) 12 times while the panel majority provided this feedback 68 times. Additionally, the most common AI feedback for incorrect squats was “neck extended too far upwards”, a correction provided 90 times by the AI and only 2 times by the panel. These under-corrections and over-corrections may be sources of diagnostic error that explain the equivocal PPV, NPV, and low specificity despite satisfactory sensitivity. Another source of diagnostic discrepancy was the panel’s ability to identify eight additional corrections beyond the standardized list. These include “incomplete extension on ascent” and “asymmetrical weightbearing” (see Fig. 3). The AI may be limited currently in its capacity to identify subtle changes in three-dimensional space in comparison to the evaluators.

Inter-rater reliability merits further explanation. All three evaluators provided input and established the working definition for a correct squat; received the same standardized list of corrections; performed their analyses blinded to set number and group allocation; and attained at least a decade of experience in physical therapy and exercise instruction, yet the homogeneity of evaluators was not reflected in the heterogeneity of IRR calculations. Although the three evaluators completely agreed for 294 (49%) repetitions, Light’s Kappa was 0.337 and is interpreted as minimal agreement^29^. The operating characteristics (i.e., sensitivity, specificity, PPV, NPV, and accuracy) of comparing AI with instances where the panel of evaluators had 3/3 complete agreement fall within the confidence intervals of the original test characteristics calculated with 2/3 majority agreement. Such findings suggest that the consensus derived from panel majority maintains consistency with more stringent criteria. The test characteristics for AI versus each individual evaluator are comparable with the panel majority as well.

One risk of the AI ability to detect incorrect squats is patient exercise safety. While this study population was comprised of healthy individuals, patients with specific musculoskeletal rehabilitation requirements may be more vulnerable to errors in exercise form, and thus more likely to experience insufficient improvements or injury due to improper movement. Of note, there were no adverse events in the PT or AI group and the AI intervention was well tolerated by study participants. A distinct advantage of this evolving technology is the potential for more equitable dissemination of safe exercise coaching. In metropolitan areas, gym memberships can cost 20 to 100 USD per month, which does not always include costs associated with hiring personal trainers or enrolling in group fitness classes^30^. For individuals who cannot afford or do not have access to these facilities and resources, or for those who prefer to exercise at home and in outdoor spaces with minimal equipment, the on-demand mobile app format is appealing as the AI technology advances.

## Limitations

As expected in this healthy adult population, some participants may not have been entirely naïve to the squat movement, which could have limited the ability of this intervention to demonstrate clinical improvement in squat outcomes. As the recruitment of participants was limited to academic institution affiliates, ages 20 to 35, and without any preexisting medical condition, further studies will be necessary to generalize the findings to patient populations or individuals with specific physical rehabilitation requirements. The AI and PT only evaluated squats in the sagittal plane and adding multiple views could change the accuracy of either evaluator. The low Light’s Kappa for feedback provided in the subset of incorrect squats could have been due to evaluators’ subjective interpretations of each participant’s anatomical variance; individual evaluators could have also focused on different aspects of the squat as more important at a single point in time.

## Conclusions

While there was no statistically significant association between group allocation and improved squat performance, the current iteration of AI has satisfactory ability to identify correct squat form and is well-tolerated in a healthy adult population. However, the AI has limited ability to identify incorrect squat form, which reduces its diagnostic capabilities. Specific improvements could include enhanced recognition of squat depth and spine biomechanics via anatomical subtleties in three-dimensional spatial detection. Future research studies should consider expanding population demographics to include various levels of squat familiarity for identification and improvement of squat form.

## Supplementary Information

Supplementary Information 1.

Supplementary Information 2.

## Data Availability

The datasets generated and analyzed during the current study are not publicly available to maintain privacy of participants; relevant de-identified data and statistical analyses are included in the manuscript.
