# Supplementary material for: Artificial intelligence application versus physical therapist for squat evaluation: a randomized controlled trial
Source: Sci Rep. 2021 Sep 13;11:18109. doi: 10.1038/s41598-021-97343-y (PMC8437936; doi:10.1038/s41598-021-97343-y)
Supplement: Supplementary file 2 — Supplementary Information 2. [file 41598_2021_97343_MOESM2_ESM.docx]

| **Tracking Information** | |  |
| --- | --- | --- |
| **First Submitted Date ^ICMJE^** | November 5, 2020 | |
| **First Posted Date ^ICMJE^** | November 12, 2020 | |
| **Last Update Posted Date** | November 12, 2020 | |
| **Actual Study Start Date ^ICMJE^** | December 5, 2019 | |
| **Actual Primary Completion Date** | December 30, 2019   (Final data collection date for primary outcome measure) | |
| **Current Primary Outcome Measures ^ICMJE^  (submitted: November 5, 2020)** | Number of correct squats [ Time Frame: Up to 15 minutes or completion of third set of squats ]  Post-intervention improvement in squats will be determined by the number of correct squats in the third set as compared to the first set of squats. | |
| **Original Primary Outcome Measures ^ICMJE^** | *Same as current* | |
| **Change History** | No Changes Posted | |
| **Current Secondary Outcome Measures ^ICMJE^  (submitted: November 5, 2020)** | Number of squats that are identified correctly by AI [ Time Frame: Up to 15 minutes or completion of third set of squats ]  AI identification of correct and incorrect squats will be determined by the number of squats that are identified correctly by AI as compared with independent evaluators. | |
| **Original Secondary Outcome Measures ^ICMJE^** | *Same as current* | |
| **Current Other Pre-specified Outcome Measures** | *Not Provided* | |
| **Original Other Pre-specified Outcome Measures** | *Not Provided* | |
|  | |  |
| **Descriptive Information** | |  |
| **Brief Title ^ICMJE^** | AI Versus HCP for Bodyweight Squats | |
| **Official Title ^ICMJE^** | Artificial Intelligence (AI) Mobile Application Versus Health Care Provider (HCP) for Bodyweight Squats: A Randomized, Blinded, Controlled Clinical Trial | |
| **Brief Summary** | To assess if an artificial intelligence (AI) mobile application can identify and improve bodyweight squat form in adult participants when compared to a Physical Therapist (PT). | |
| **Detailed Description** | Artificial intelligence (AI) is changing the way people can address their health needs. One such way related to physical exercise is AI-enabled exercise mobile application (digital coach), which uses motion tracking technology to monitor and provide real-time audio feedback on a person's exercise form. However, this AI technology has yet to be independently tested against an in-person evaluator (human coach) for its ability to improve exercise form. This study is a blinded randomized controlled trial comparing the ability of the digital coach (n=15) and a Physical Therapist (PT) human coach (n=15) to improve bodyweight squat form in 30 able-bodied volunteers age 20 - 35. Each volunteer performs 10 unassisted control squats, then 10 squats with assistive vocal feedback from either coach after each repetition, and finally 10 more unassisted test squats, all squats video-recorded. Three independent video evaluators count the number of correct squat repetitions completed by volunteers before and after intervention by the different coaches. This project is important to validate the digital coach compared to a PT human coach in a small population using a bodyweight squat for its wide applicability to daily movement patterns. | |
| **Study Type ^ICMJE^** | Interventional | |
| **Study Phase ^ICMJE^** | Not Applicable | |
| **Study Design ^ICMJE^** | Allocation: Randomized Intervention Model: Parallel Assignment Masking: Single (Outcomes Assessor) Primary Purpose: Other | |
| **Condition ^ICMJE^** | Squat Form | |
| **Intervention ^ICMJE^** | - Other: Artificial Intelligence Feedback   AI mobile application provides feedback to participants randomized to artificial intelligence group.   - Other: Physical Therapist Feedback   PT provides feedback to participants randomized to physical therapist group. | |
| **Study Arms ^ICMJE^** | - Experimental: Artificial Intelligence (AI) Group   To determine baseline ability and serve as their own control, participants in both groups performed 10 bodyweight squat "control" repetitions without feedback followed by one minute of rest. Those in the AI group then performed 10 more "practice" repetitions with real-time audiovisual feedback from the app followed by one minute of rest. The AI's design provided one piece of feedback, if necessary, with a vocal statement and on-screen video per repetition (e.g. when a participant performed a squat repetition with their neck flexed downward, AI suggested keeping their head up with on-screen instruction). Participants in both groups then performed 10 "test" repetitions without feedback followed by one minute of rest.  Intervention: Other: Artificial Intelligence Feedback   - Active Comparator: Physical Therapist Group   To determine baseline ability and serve as their own control, participants in both groups performed 10 bodyweight squat "control" repetitions without feedback followed by one minute of rest. Those in the PT group (n=15) also performed 10 "practice" repetitions with one piece of feedback per repetition, if necessary, from the PT followed by one minute of rest. Participants in both groups then performed 10 "test" repetitions without feedback followed by one minute of rest.  Intervention: Other: Physical Therapist Feedback | |
| **Publications *** | *Not Provided* | |
| ***   Includes publications given by the data provider as well as publications identified by ClinicalTrials.gov Identifier (NCT Number) in Medline.** | |  |
|  | |  |
| **Recruitment Information** | |  |
| **Recruitment Status ^ICMJE^** | Completed | |
| **Actual Enrollment ^ICMJE^  (submitted: November 5, 2020)** | 30 | |
| **Original Actual Enrollment ^ICMJE^** | *Same as current* | |
| **Actual Study Completion Date ^ICMJE^** | December 30, 2019 | |
| **Actual Primary Completion Date** | December 30, 2019   (Final data collection date for primary outcome measure) | |
| **Eligibility Criteria ^ICMJE^** | Inclusion Criteria:   - Columbia University affiliate - Aged 20 to 35 years - Able to perform moderate bodyweight exercise for 10 minutes | |
| **Sex/Gender ^ICMJE^** | \| Sexes Eligible for Study: \| All \| \| --- \| --- \| | |
| **Ages ^ICMJE^** | 20 Years to 35 Years   (Adult) | |
| **Accepts Healthy Volunteers ^ICMJE^** | Yes | |
| **Contacts ^ICMJE^** | *Contact information is only displayed when the study is recruiting subjects* | |
| **Listed Location Countries ^ICMJE^** | United States | |
| **Removed Location Countries** |  | |
|  | |  |
| **Administrative Information** | |  |
| **NCT Number ^ICMJE^** | NCT04624594 | |
| **Other Study ID Numbers ^ICMJE^** | AAAS7301 | |
| **Has Data Monitoring Committee** | No | |
| **U.S. FDA-regulated Product** | \| Studies a U.S. FDA-regulated Drug Product: \| No \| \| --- \| --- \| \| Studies a U.S. FDA-regulated Device Product: \| No \| | |
| **IPD Sharing Statement ^ICMJE^** | \| Plan to Share IPD: \| No \| \| --- \| --- \| \| Plan Description: \| Individual participant data is not shared with other researchers \| | |
| **Responsible Party** | Columbia University | |
| **Study Sponsor ^ICMJE^** | Columbia University | |
| **Collaborators ^ICMJE^** | National Medical Fellowships | |
| **Investigators ^ICMJE^** | \| Principal Investigator: \| Sunil K. Agrawal, PhD \| Columbia University \| \| --- \| --- \| --- \| | |
| **PRS Account** | Columbia University | |
| **Verification Date** | November 2020 | |
| **^ICMJE^    Data element required by the**[**International Committee of Medical Journal Editors**](http://www.icmje.org/recommendations/browse/publishing-and-editorial-issues/clinical-trial-registration.html)**and the**[**World Health Organization ICTRP**](https://www.who.int/clinical-trials-registry-platform) | |  |
